# Transcriptomic analysis supports similar functional roles for the two thymuses of the tammar wallaby

**DOI:** 10.1186/1471-2164-12-420

**Published:** 2011-08-19

**Authors:** Emily SW Wong, Anthony T Papenfuss, Andreas Heger, Arthur L Hsu, Chris P Ponting, Robert D Miller, Jane C Fenelon, Marilyn B Renfree, Richard A Gibbs, Katherine Belov

**Affiliations:** 1 Faculty of Veterinary Sciences, University of Sydney, Sydney, NSW 2006, Australia; 2 Bioinformatics Division, The Walter and Eliza Hall Institute for Medical Research, Parkville, Victoria 3050, Australia; 3 Medical Research Council Functional Genomics Unit, Department of Physiology, Anatomy and Genetics, University of Oxford, Oxford, UK; 4 Center for Evolutionary and Theoretical Immunology, Department of Biology, University of New Mexico, Albuquerque, NM, USA; 5 ARC Centre of Excellence in Kangaroo Genomics, Department of Zoology, University of Melbourne, Victoria 3010, Australia; 6 Department of Molecular and Human Genetics, Human Genome Sequencing Center, Baylor College of Medicine, One Baylor Plaza, Houston, TX 77030, USA

## Abstract

**Background:**

The thymus plays a critical role in the development and maturation of T-cells. Humans have a single thoracic thymus and presence of a second thymus is considered an anomaly. However, many vertebrates have multiple thymuses. The tammar wallaby has two thymuses: a thoracic thymus (typically found in all mammals) and a dominant cervical thymus. Researchers have known about the presence of the two wallaby thymuses since the 1800s, but no genome-wide research has been carried out into possible functional differences between the two thymic tissues. Here, we used pyrosequencing to compare the transcriptomes of a cervical and thoracic thymus from a single 178 day old tammar wallaby.

**Results:**

We show that both the tammar thoracic and the cervical thymuses displayed gene expression profiles consistent with roles in T-cell development. Both thymuses expressed genes that mediate distinct phases of T-cells differentiation, including the initial commitment of blood stem cells to the T-lineage, the generation of T-cell receptor diversity and development of thymic epithelial cells. Crucial immune genes, such as chemokines were also present. Comparable patterns of expression of non-coding RNAs were seen. 67 genes differentially expressed between the two thymuses were detected, and the possible significance of these results are discussed.

**Conclusion:**

This is the first study comparing the transcriptomes of two thymuses from a single individual. Our finding supports that both thymuses are functionally equivalent and drive T-cell development. These results are an important first step in the understanding of the genetic processes that govern marsupial immunity, and also allow us to begin to trace the evolution of the mammalian immune system.

## Background

The thymus is a site of T-cell differentiation and maturation. First discovered by Galen (130-200 AD), it is a primary lymphoid organ with a critical role in development of the immune system. Its major function is to eliminate developing T-cells (thymoctyes) whose antigen receptor can bind self antigens and have the potential to cause autoimmune disease [1,2]. The thymus is comprised of two distinct regions, the cortex and the medulla, both of which produce thymic epithelial cells. On entering the thymus, thymocytes undergo clonal proliferation, lineage commitment and selection for a T-cell receptor (TCR) that can interact with self Major Histocompatibility Complex molecules. This is primarily under the control of cortical thymic epithelial cells [2]. To ensure that autoreactive thymocytes are eliminated, a diversity of self-antigens, representing a wide range of tissue types, is presented to thymocytes by medullary thymic epithelial cells [3]. Therefore the medullary epithelium must synthesize a large fraction of the potential proteome of the individual to display as self. Autoreactive thymocytes are eliminated by apoptosis. In total, 95-99% of thymocytes are eliminated this way, leaving only a 1-5% pool of functional T-cells that eventually enter peripheral circulation as mature T-cells [4,5].

All jawed vertebrates possess a thymus. Eutherian mammals, such as humans and dogs, usually possess a single thoracic thymus. A thymus in the neck of humans (called a cervical thymus) is considered to be an often asymptomatic pathological condition whereby the thoracic thymus has failed to descend to its proper mediastinal location [6,7]. However, the number of thymuses per animal, the anatomical position and structure of the thymic lobes and the exact developmental origin of the thymus all differ markedly among species [7]. Multiple thymuses are common in cold-blooded vertebrates, with five pairs found in shark, four in caecilian amphibians, three in salamanders and one found in cartilaginous fish [7]. In other eutherian mammals such as sheep, cattle, goat and horse, the paired thymuses consist of a distinct cervical and thoracic part; the stem of the thymus connecting the two sections typically disappears shortly after gestation [8,9]. Certain strains of mice possess an additional thymic organ in the cervical area, with distinct cortical and medullary regions that support positive and negative selection of thymocytes and export mature thymocytes to the circulation [10,11].

The discovery of cervical thymuses in marsupials in the 1800s provided some of the earliest documentation of the cervical thymus in any mammal [12-15]. It is now established that diprotodont marsupial species, including kangaroo, wallabies and possums, typically possess both cervical and thoracic thymuses [16-21]. The exception to this is the koala (*Phascolarctos cinereus*), in which a lone thoracic thymus is more commonly found [20]. Early studies focused on topological and anatomical descriptions of the thymuses including details on their organogenesis and relative growth rate [12-15,18,19,22]. Later, histological studies were used to determine the time of T-cell maturation [23-27].

Marsupials deliver highly altricial young that complete their development post-natally [28] so their two pairs of thymuses develop after birth while the young is in the pouch [29,30], making them ideal model organisms to study the development of the immune system. At birth, marsupials lack functional lympoid tissues [27,30,31] and humoral immune competence (mediated by antibodies). Cell-mediated immune responses (associated with T-cells) develop during pouch life (reviewed by [32]). In the tammar wallaby (*Macropus eugenii*), T-lymphocytes are observed at Day 2 in the cervical thymus [24] and CD3+ mature T-cells are first detected in thymic tissue on Day 12 postpartum [26]. This coincides with the differentiation of the cervical thymus into a rudimentary cortex and medulla [24,26]. The thymuses are largest from mid-pouch life, and adult lymphoid tissue structure is seen at approximately Day 120 [24,26], which is why we chose 178 day old tissue for this experiment. At maturity, distinct cortical and medullary regions and Hassall's corpuscles, groups of specialized epithelial cells localized to the medulla that direct maturation of a lineage of T-regulatory cells, are present in both thymuses [24,26,33].

The marsupial cervical thymus reaches enormous proportions during pouch life when it obscures all other cervical glands [17]. It is larger than the thoracic thymus at all development stages [17,34]. In a 150-day-old tammar wallaby pouch young the thoracic thymus weighs 94 mg, a mere 7% of the cervical thymus which weighs 1,400 mg or 0.7% of total body weight [17]. At Day 120, the thoracic thymus is not as multi-lobed as earlier stages of the cervical thymus [24].

Several findings support the notion that both organs have a similar, if not identical role. Mature T-cells are present in both thymuses in the tammar and in another wallaby, the quokka (*Setonix brachyurus*) throughout development [24,26]. Onset of humoral responses are delayed upon removal of the cervical thymus and total thymectomy further delays, but does not abrogate, the ability of quokkas to mount a T-cell dependent humoral immune response [34]. Notably, neonatally thymectomized quokkas have a significantly reduced lifespan [35].

Here, we compare gene expression profiles in the cervical and thoracic thymus of a marsupial, the tammar wallaby. This is the first transcriptomic study of both the cervical and thoracic thymus from any species. We predicted that both organs would display similar transcriptomic profiles and express genes that are critical for thymic function.

## Results and discussion

RNA from cervical and thoracic thymic tissues from a 178-day-old tammar wallaby pouch young was extracted and pyrosequenced using the Roche 454 platform. 758,062 reads with an average read length of 184 bases were generated. Checks on sequencing biases are provided as additional file 1 and additional file 2. Annotated gene sequences are available at http://bioinf.wehi.edu.au/tammar. Full datasets are stored in the NCBI Short Reads Archive, accessions SRX019250 and SRX019249.

### Gene abundance analysis

Reads were aligned to the wallaby genome (version 1.0) using BLASTN. 87% of reads aligned to the genome. Reads that aligned well to two or more regions of the genome (28%) were filtered out. This might have removed members of closely related gene families. Consequently, we also analysed reads without filtering. The two analyses resulted in similar results (data not shown) and had no effect on our main conclusions.

Based on alignment with the wallaby Ensembl gene build, 6,175 genes are expressed in either of the two thymuses, with 4,642 being expressed in both thymuses. However, as is commonly observed in transcriptome analyses, the majority (79%) of reads failed to align to a predicted Ensembl gene. These reads are likely to represent 5' or 3' untranslated regions (UTRs) not present in Ensembl gene models, or non-protein-coding transcripts or incorrectly predicted exons.

To increase the confidence of our analysis, we aligned the tammar reads against the higher quality opossum genome (a 7× coverage marsupial genome). A similar number of genes were identified in either of the two thymuses (6,060 genes). More reads aligned to intergenic sequence in the tammar assembly (79%) compared with the opossum assembly (41%). This may reflect the expression of regions poorly conserved between the two marsupial genomes and currently unannotated genes in tammar.

### Further thymic gene identification

**Figure 1:**
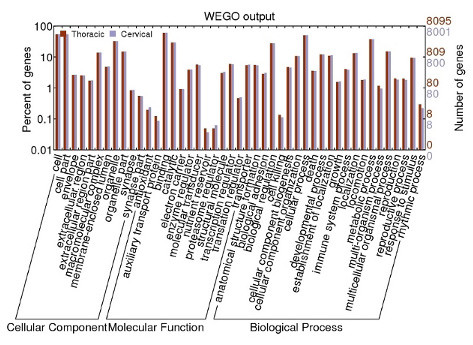
**Gene numbers from each thymus corresponding to a range of GO categories**.

In order to improve the sensitivity of our sequence database searches, we assembled reads pooled from both tissues. Since most genes show similar expression levels in the two tissues (Figure 1), on average, this doubles the number of reads mapped to each gene (compared to separate assemblies) and thus will tend to provide longer transcript contigs. The resultant assembly of reads from both thymuses produced 36,591 transcript contigs. We used these contigs together with unassembled reads to identified 3,148 additional genes in the wallaby genome assembly.

Many immune genes are likely to be left unannotated by automated annotation pipelines, such as Ensembl, due to their more rapid evolution. To increase the sensitivity of immune gene identification we used manually curated opossum (*Monodelphis domestica*) immune gene lists [36,37] of 1,549 opossum genes. Less than a third (463) of these were annotated by the Ensembl opossum geneset. Using this curated geneset we were able to identify the expression of an additional 222 immune genes in the wallaby, adding to the 356 expressed wallaby immune genes that were already annotated by Ensembl http://bioinf.wehi.edu.au/tammar. We report the expression of 34 cytokines and their receptors (10 chemokines, 22 interleukins and 2 interferons), 22 natural killer cell receptors (20 Leukocyte Receptor Complex (LRC) genes and two Natural Killer Complex (NKC) genes), three antimicrobial peptides (two beta-defensins and one cathelicidin), post-switch immunoglobulin isotypes IgA and IgG and *CD4 *and *CD8 *T-cell markers. We expect that unidentified immune genes are either unsequenced at this sequencing depth, are not expressed in the thymuses or are too divergent to identify using our search method.

### Thymic gene function

We identified a total of 9,545 genes in the two tammar thymus pairs. Within this dataset, we identified transcription factors and signalling molecules that control the development of haematopoietic stem cells committed to T-cells lineages, including *GATA3, IKZF1, RUNX3, LEF1, JAG1, NOTCH1, IL-7R, MYB, HEB *and *E2A *(reviewed in [38]) (Table 1). These molecules are expressed during early T-cell development and are essential for the promotion of hematopoietic stem cells to the T-lineage. *GATA3 *and *RUNX3 *play important roles in CD4- and CD8-lineage choice, a process that occurs at later stages of T-cell development [38-42]. Together, these proteins form complex interactions which promote thymocyte differentiation. For example, *HEB *and *E2A *fall into a group of class I helix-loop-helix proteins, also known as E proteins. As heterodimers these activate the pre-T-cell antigen receptor alpha (*PTCRA*), an invariant T-cell receptor alpha chain that forms the pre-TCR essential for alpha-beta lineage T-cell differentiation [43]. One of the two isoforms of *E2A*, *E47*, also regulates the gene rearrangement proteins, *RAG1/2*, which are initiators of T-cell receptor gene recombination, a key step in the generation of receptor diversity, in early T-lymphocytes development [43,44]. Furthermore, *E2A *also acts in concert with Notch signalling, to promote differentiation of T-cells and to suppress progenitor cell commitment to NK and myeloid cell fates [45]. The Notch signalling pathway is a critical pathway for the initiation of T cell development [46-48]. Induced deletion of *NOTCH1*, a transmembrane transcription activator, in mouse results in an early stage blockage in T-cell development [47]. Notch signalling induced by Jagged1 (*JAG1*) plays a critical role in thymocyte cell-fate determination [49]. Notch signals have also been shown to cooperate with the *IL-7R *pathway to sustain *IL-7R *expression in proliferating thymocytes [50]. Signalling of *IL-7R *has a prominent role in thymocyte proliferation in early T-cell development [51]. Accordingly, *IL-7R *is tightly regulated in later stage committed T-cells [52] and contributes to regulatory T-cell development and homeostasis outside the thymus [53]. Notably, key genes involved in mouse cervical and thoracic thymopoiesis: *RAG1, RAG2 *and *DNTT *[10], were identified in both tammar thymuses.

**Table 1:** Human gene symbol and its corresponding description of critical thymic genes transcribed in both wallaby thymuses.

HGNC Symbol	Description
LTBR	Tumor necrosis factor receptor superfamily member 3

NOTCH1	Transmembrane receptor for transcriptional activation

JAG1	NOTCH1 ligand

IL7R	Interleukin-7 receptor subunit alpha precursor

GATA3	Trans-acting T-cell-specific transcription factor

XRCC4	DNA repair protein XRCC4

TRAF6	TNF receptor-associated factor 6

RAG1	V(D)J recombination-activating protein 1

RAG2	V(D)J recombination-activating protein 2

LEF1	Lymphoid enhancer-binding factor 1

IKZF1	DNA-binding protein Ikaros

RUNX3	Runt-related transcription factor 3

DNTT	DNA nucleotidylexotransferase (Terminal addition enzyme)

MYB	Myb proto-oncogene protein

GFI1	Zinc finger protein

TP63	Tumor protein 63 (p63)

E2A	E-protein gene

HEB	E-protein gene

We detected the presence of key chemokine receptors, *CCR7 *and *CCR9*, in both wallaby thymuses. Chemokines are small proteins that are able to induce chemotaxis, and in the thymus, they help dictate the movement of T-cells from initial precursor cell recruitment through to mature thymocyte export. Chemokine expression therefore must be controlled and this is reflected through the unique chemokine expression profiles in different thymic cell types [54]. Both *CCR7 *and *CCR9 *maintain an important role during T-cell development, ensuring that developing thymocytes are positioned at specific thymic microenvironments to support T-cell differentiation [55]. Movement of thymocyte into the medulla from the cortex after positive selection is signalled by *CCL7 *[56], with premature positioning of CD4+CD8+ thymocytes into the medulla impairing T-cell development [57]. Similarly, *CCL9 *is responsible the movement of immature T-cells to the thymic subcapsular zone [58].

Other genes essential in thymocyte development include *TRAF6*, *TP63 *and *LTBR*. These genes are necessary for the development and maintenance of normal thymic architecture. *TRAF6*, a signal inducer of the NK-kB pathway, is necessary for the organization of medullary thymic epithelial cells (mTECs), and *TP63 *is required for normal epithelial development [59-61]. Cross-talk between mTECs and thymocytes is essential for mTEC differentiation. This exchange is mediated by the lymphotoxin beta receptor (*LTBR*) [62].

All major classes of T-cell receptor chains were expressed in both tammar thymuses (Table 2). The ability of T-cells to recognize pathogens is mediated by their T-cell receptors. T-cell receptors have been detected in opossum neonates as early as Day 1 [63], although T-cell dependent cellular immune responses are not observed until the second week of development [64]. TCR chains are classified according to their constant regions-alpha (*TRAC*), beta (*TRBC*), gamma (*TRGC*), delta (*TRDC*) and mu (*TRMC*) [65,66]. The novel T-cell receptor mu (TRM) that was first discovered in marsupials and has not been found in eutherians [66,67]. In vertebrates, each T-cell bears a unique T-cell receptor that is specific to a limited set of peptide and major histocompatibility complex (MHC) combinations [65]. The T-cell receptors (TCR) are formed from disulfide-linked heterodimers which are composed of either an alpha and a beta chain or a gamma and a delta chain. Alpha-beta T-cell receptors recognize MHC molecules and undergo a process of ligand-driven positive and negative selection leading to maturation to T-helper cells or T-killer cells. Delta-gamma T-cells bind to a different set of ligands and do not require antigen-based selection for maturation [68,69]. To be effective in recognizing a wide variety of antigens, a diverse repertoire of TCR chains are generated through a process known as somatic recombination involving variable(V), diversity(D) and joining(J) genomic segments. Tammar transcripts displayed evidence of somatic recombination.

**Table 2:** Genomic locations in the tammar assembly and number of reads across both thymuses for constant regions of T-cell receptors.

	Genomic scaffold	Read count
TRBC	45360	85

	544743	30

	29035	25

	6676	2

TRDC	14893 on chromosome 7p	1

TRAC	32403 on chromosome 7p	3

TRGC	6369	1

	340996	1

TRMC	21414	1

	73733	1

	92637	6

	52643	2

TRA/D have been mapped to chromosome 7p [74].

Four TCR beta chain constant (*TRBC*) genes showed highly variable number of transcripts (Table 2). This may be due to selective preference for certain *TRBC *genes over others. Four *TRBC *genes exist in opossum, but both human and mouse TRB loci comprise of just two TRB constant genes [67,70]. We identified five putative *TRBC *genes in tammar genome, one of which we did not have any expression data for, and suggest that up to five TRB genes may potentially exist in tammar.

One *TRDC *transcript with 91% identity with cloned wallaby *TRDC *transcript (Accession: AAP72021) was identified, with mismatches between the sequences likely to be due to either allelic differences or sequencing errors. The TRA/D locus spans ≥ 1 Mb in the opossum, human and mouse genomes with the TRD locus nested within the TRA locus [67,70]. The presence of a single constant region for each chain is consistent with known TRA/D genomic structure in all vertebrates.

Two wallaby TRG constant regions (*TRGC*) genes were found. *TRGC *regions vary in number between species with human and mouse both possessing a single TRG locus which contains two and four *TRGC *genes, respectively, which are arranged in tandem cassettes [71,72]. The cow and sheep have two TRG loci each, which containing variable numbers of constant regions [73]. Only one TRG locus exists in the opossum, comprising a single constant gene, arranged in a translocon organization with V and J regions adjacent to one another [67]. Both scaffolds reside on chromosome 3p [74] but it remains to be determined whether the two wallaby TRGC genes localize to the same locus, like in human and mouse, or to different loci, as in ruminants. *TRMC *is also expressed at comparable levels between the two thymuses [66].

Both thymuses express transcripts involved in the process of somatic recombination (VDJ recombination) which is necessary for T-cell receptor generation. These include recombination activating gene-1 (*RAG1*), *RAG2*, DNA-dependent protein kinase, Artemis, DNA ligase IV and *XRCC4*. The initiation of VDJ recombination involves the lymphoid-specific proteins, *RAG1 *and *RAG2*, which introduce double-strand breaks in signal sequence adjacent to coding segments (reviewed in [75]). Broken ends bind to DNA-dependent protein kinase which combines with Artemis to break the hairpin structure introduced by the RAG proteins [76,77]. Finally, the ligation of two ends is mediated by DNA ligase IV which forms a heteromultimer with *XRCC4*, a protein that serves to enhance the joining activity [78-80].

Due to the low sequence coverage of the transcriptomes, the absence of some genes in our database was expected. We did not expect to see lowly expressed genes, and did not find thymic stromal lymphopoietin transcripts (*TSLP*). In humans it promotes the expression of *CD80 *and *CD85 *in dendritic cells, which in turn induce the expansion differentiation of groups of thymic T-cells into regulatory T-cells [33]. *TSLP *is expressed by Hassall's corpuscles, a distinct group of medullary TECs. Hassall's corpuscles are observed in the wallaby cervical and thoracic thymuses by days 21 and 30 respectively. Hence, we expected to identify *TSLP *in a 178 day old wallaby thymus. *TSLP *exhibit a high level of sequence divergence. We identified the gene using a hidden Markov model (HMM) profile, but not the transcript.

### Non-protein-coding RNAs

Given the abundance of transcripts that did not map to gene models, we performed some analyses on non-coding RNA transcripts identified in both thymuses. 3,188 putative non-coding contigs were conserved between the opossum and tammar wallaby. To identify putative non-coding RNAs (ncRNAs), all contigs were analysed using the program CPC [81]. Contigs were considered non-coding provided that they do not encode open reading frames and are not homologous to known protein sequences. 83,294 potential non-coding elements were identified. We subsequently aligned all potential non-coding RNAs to the opossum genome, resulting in 12,703 mapped contigs. To avoid the misannotation of UTRs as non-coding RNAs, we examined 3,524 contigs that aligned to a distance of 100 kb or greater to their adjacent Ensembl gene. Notably, approximately half of all predicted non-coding structural elements in human are located over 10 kb away from any known gene, although these have not been verified experimentally [82]. These contigs were searched against a comprehensive database of human UTR regions, UTRfull [83]. 3,188 reads remained after removal of sequences aligning to known UTRs with E-values < 10^-3 ^The annotation of the resultant ncRNA candidates against non-coding databases, fRNAdb [84] and RFAM [85], identified 101 non-coding elements including 52 conserved non-coding structures that were determined by RNAz [86] and EvoFold [87] (Table 3). Wallaby non-coding contigs were located in the genome closest to genes that are involved in transcriptional regulation (P = 4.3 × 10^-5 ^Fisher exact test with Bonferroni correction). This is consistent with genomic observations in vertebrates, where conserved non-coding elements tend to be located adjacent to transcriptional regulators, suggesting distant regulatory functions [88-91]. Evolutionarily conserved non-coding elements show a level of synteny similar to coding genes [92], and behave as transcriptional enhancers *in vivo *[91,93]. Our results represent the first set of candidate ncRNAs conserved between marsupials. To examine whether any overlapping non-coding elements exist between our contigs and a highly conserved *Fugu *dataset [91], we searched against the conserved pufferfish sequences and retrieved four tammar contigs. Two of these were similar to piRNAs and one matched a known transposable L1 LINE 3' element.

**Table 3:** Classification of putative functional non-coding RNAs.

NcRNA type	Number of NcRNAs identified
piRNA	72

tRNA	16

miRNA	13

Ribosomal RNA	6

Unclassified small RNAs	6

L1 LINE 3' elements	2

RNAz non-coding structures	28

EvoFold non-coding structures	24

The miRNAs map to seven separate seed regions.

### Differential gene expression between cervical and thoracic thymuses

**Figure 2:**
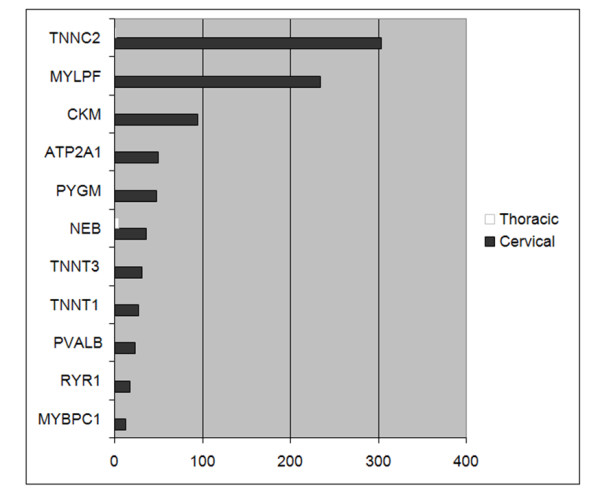
**Read counts for differentially expressed muscle genes**.

Of all genes identified in the two thymuses, only 67 genes were expressed at significantly different levels in the two thymuses (*p *< 0.05 Fisher exact test with FDR correction). The cervical thymus over-expressed a group of genes with strong associations to muscle structure, assembly and contraction. Testing for enrichment of GO terms in genes more highly expressed in the cervical thymus, identified nine over-represented terms (*p *< 0.05; Fisher exact test with Bonferroni correction). Of these, eight GO terms were linked either directly or indirectly to muscle fibres (*p *= 2.8 × 10^-6^; Fisher exact test with Bonferroni correction) (Table 4; Figure 2). *ATP2A1 *together with ryanodine receptor, *RYR1*, and muscle glycogen phosphorylase (*PYGM*) are typically found in the sarcoplasmic reticulum, a subtype of smooth endoplasmic reticulum found in muscle fibres whose function is to store and release calcium ions. Both troponin T and creatine kinase muscle (*CKM*) were over-expressed by the cervical thymus. Notably, these genes are also highly expressed in human myoid cell lines [94].

**Table 4:** Names of genes differentially expressed between the two thymuses linked to GO term 'muscle fibres'.

Name	Gene symbol
sarcoplasmic reticulum calcium ATPase 1	*ATP2A1*

myosin binding protein C, slow type	*MYBPC1*

myosin regulatory light chain 2	*MYLPF*

nebulin	*NEB*

sarcoplasmic reticulum calcium release channel receptor	*RYR1*

fast skeletal muscle troponin C	*TNNC2*

slow skeletal muscle troponin T	*TNNT1*

fast skeletal muscle troponin T	*TNNT3*

Thymuses are known to carry cells containing striated myofibrils which closely resemble skeletal or cardiac muscle fibers. Known as myoid cells, these cells are conserved throughout vertebrate evolution, yet, prior to this study, myoid cells have not been identified in the marsupial thymus [17,20,23,24,26,95]. However, they have been described in vast numbers of eutherian, bird, reptile, amphibian, and teleost species [96-98]. Myoid cells appear to be abundant in non-eutherian mammals and younger eutherian animals. In birds and reptiles, however, they appear to increase in number with age. Reptilian myoid cells numbers vary depending on season [96]. Furthermore, they display a wide spectrum of muscle cell developmental stages [96]. Although functional studies on myoid cells are lacking over all phyla, myoid cells have been shown in humans *in vitro *to have protective effects against apoptosis and to increase the population of CD4+ thymocytes [99]. In the medullary region of human embryos, there is an increase in the number of myoid cells along with a corresponding increase in amount of medullary myosin in the late developmental stages of the embryos [100]. It has been suggested that this may reflect the demands of the growing fetus for mature T lymphocytes [100]. If this is true, it is conceivable that such a demand may be heightened by the immunologically immature state of marsupials at birth.

Several genes, identified in the cervical thymus, are integral to muscle contraction. Given that myoid cells are known to spontaneously contract *in vitro *this may aid the movement of thymocytes *in vivo *[100,101]. The Ca^2+ ^transporter molecule, *ATP2A*, and the troponin proteins, are involved in the Ca^2+ ^binding pathway that leads to muscle contraction [102,103]. These were over-expressed in the cervical thymus.

The preponderance of muscle related genes in the cervical thymus is unlikely to be due to the expression of a diverse array of tissue-specific genes in medullary thymic epithelial cells (mTECs) during negative selection [104] even though numerous tissue-specific antigens are known to be expressed in mTEC cells. The expression of tissue specific antigens allows potential auto-immune T cells to be detected and subsequently destroyed. Promiscuously expressed tissue-specific antigens tend to be colocalized in genomic clusters and their expression appears to be primarily controlled by pathways involving the transcriptional regulator, *AIRE *[105,106]. But the differentially expressed muscle genes in tammar thymuses do not appear to be clustered in the genome (based on the opossum genome) and it is unlikely that a muscle-specific group of genes would be expressed in a concerted fashion by mTECs in one thymus but not the other.

Parvalbumin alpha (*PVALB*) was also expressed more highly in the cervical than in the thoracic thymus. *PVALB *is a calcium-binding protein found in skeletal myofibrils and GABA-producing neurons, with a role in the relaxation of muscle after contraction [107,108]. Interestingly, a paralog of *PVALB*, avian thymic hormone (*ATH*), promotes chicken thymocyte maturation [109,110]. According to Ensembl annotations, orthologs of *ATH *are present in marsupials and monotremes but are missing in eutherian species. This not only suggests that *ATH *was lost in the eutherian lineage but also indicates a possible role of *ATH *in T-cell development in marsupials and monotremes, as in chicken. However, transcripts of *ATH *were not detected in either tammar thymus transcriptome, probably due to the low coverage of the transcriptome. Tammar oncomoulin (*OCM*) transcripts were detected in the thoracic thymus. *OCM *is also a paralog of *PVALB *and functions as an oncodevelopmental protein in human. Although human oncomodulin is mainly expressed in early embryonic cells, chicken oncomodulin-like protein expression is largely localized to the thymus [111]. Further studies are required to ascertain whether wallaby *OCM *is expressed more broadly and to determine whether it has an immunomodulary role in the thymus. *ATH *and *OCM *are located in close proximity of one another and show conserved synteny in chicken and opossum genomes. Their genomic position remains to be determined in the tammar due to short contig lengths.

### Possible explanations for differential expression

Differences in gene expression between the two thymuses may be explained in several ways. Firstly, differences may merely be reflective of uneven sequencing of thymic compartments. Thymic anatomical microenvironments are unique and molecular gradients control T-cell development [112]. Eutherian myoid cells localize in the thymic medulla, although occasional expression in the corticomedullary junction has been observed [113,114]. Although we have sequenced equivalent amounts of cDNA from both thymuses, it is possible that more thymic epithelial cells and thymocytes were harvested in the thoracic thymus, whilst a greater number of myoid cells were sampled in the cervical. Yet, we cannot dismiss that the cervical thymus may indeed contain a greater proportion of myoid cells than the thoracic thymus. In human, a correlation exists between the abundance of myoid cells with fetal development time [100]. In marsupials, this is supported by the fact that the cervical thymus reaches functional maturity earlier than the thoracic which may result in differential counts of myoid cells. We did not observe other variations in expression that could clearly be explained by developmental differences between the two thymuses. The higher expression of certain T-cell developmentally-associated genes in the thoracic thymus (*CD74*, *CD3G *and *NOTCH1*) may be indicative of some differences in function between the thymuses. However, this is highly speculative at this early stage as sampling bias cannot be ruled out.

### Tissue-specific antigens

Variation between tissue-restricted antigens expressed by multiple thymuses presents a potential susceptibility factor for autoimmunity. In mouse, cervical and thoracic thymuses have shown to express varying amounts of self-antigens [11]. In light of this, we compared 211 genes determined to be regulated by *Aire *in mouse medullary thymic epithelial cells [115] to tammar gene expression. In human and mouse, *Aire *regulates autoimmunity by promoting the expression of a range of tissue-restricted antigens in medullary epithelial cells.

Similar levels of self-antigens are expressed by the two tammar thymuses. Only one tissue-specific antigen, collagen type 1A (*COL1A1*), was regulated by *Aire *in mouse medullary thymic epithelial cells [116] and was differentially expressed between the two wallaby thymuses. However, given that our data represent a collection of all thymic cell types, *COL1A1 *may not be predominantly expressed by medullary epithelial cells. *COL1A1 *is a major component of muscle fibres [117]. In the context of differential co-expression of other muscle genes, variation of *COL1A1 *expression may more likely be attributed to myoid cell differences rather than selection for self-tolerance by medullary epithelial cells.

### Summary

Our results indicate that both marsupial thymuses are largely functionally equivalent. Both thymuses expressed genes that support thymic differentiation and function. These include transcripts encoding proteins with critical roles in directing thymic environment development (e.g. *TRAF6*, *TP63 *and *LTBR*) and T-cell lineage differentiation (e.g. *IL-7R, NOTCH1*, *GATA3*, *SPI1*, *IKZF1*). All T-cell receptors were expressed. In addition, key genes involved in mouse cervical and thoracic thymopoiesis were identified in both tammar wallaby thymuses. A relatively small number of genes were differentially expressed between the thymuses but these differences may be attributed to sampling artefacts. Notably, gene networks of known thymic function were not differentially expressed. The highly similar transcriptomic landscape of both tammar thymuses suggests that multiple thymuses in other species are also likely to show similar patterns of gene expression.

We have shown that both wallaby thymuses express similar levels of self-antigens. It is feasible that presence of multiple thymuses differentially selecting for self-tolerant T cells may result in autoimmunity if the two organs are projecting different versions of self [11]. In the mouse, variability in autoantigen expression has been reported [11], but we did not detect any differences in self-antigen expression between the two thymuses in the tammar wallaby.

The presence of a cervical thymus in the wallaby may be an evolutionary modification that allows rapid post-natal development of immunocompetence. Wallabies are born without immunological tissues and need to develop an immune system quickly to survive pathogen challenge in a closed pouch [27]. The size of the thoracic thymus is restricted by the size of the ribcage, while the thymus in the neck is able to grow unrestricted and allows earlier development of functional T-cells [118].

## Conclusion

This is the first study to compare gene expression in the two thymuses of a marsupial, or in fact, any species. Both tammar wallaby thymuses have largely identical roles in T-cell development and maturation and possess basic thymic functions that are comparable to human and mouse. Crucial genes involved in T-cell differentiation, positive and negative selection of T-cells, immature T-cell proliferation, as well as T-cell receptor signalling are present. Our findings provide a springboard for further research into the development of the marsupial immune system and the evolution of the mammalian immune system.

## Methods

### Library construction and sequencing

Tissues from both thymuses were harvested from a 178 day-old tammar wallaby pouch young of Kangaroo Island, South Australian origin that was killed for another project approved by the University of Melbourne Animal Experimentation Ethics Committees. cDNA libraries were generated from the samples and sequenced at the Human Genome Sequencing Center at the Baylor College of Medicine, Houston, Texas, USA, using the Roche 454 Genome Sequencer FLX System. Two runs were completed with each run comprising of half runs of each thymus.

### Reads analysis

Individual reads were assigned to gene models based on both the Ensembl wallaby and opossum genesets (Release 56). The opossum geneset is the highest quality genome of any marsupial to date and provides a useful quality comparison to the low coverage wallaby genome. A similar numbers of protein-coding genes are predicted by Ensembl between the two dataset (15,280 in wallaby and 19,466 in opossum). Reads were filtered and aligned to the *Macropus eugenii *1.0 assembly using GMAP [119] and to the *Monodelphis domestica *(Mondom5) genome assembly using GMAP [119] and BLASTN [120]. The filtering stage involves removing reads mapping to more than one area with < 10% difference in scores, removing reads with less than 30 bases aligning and those with alignment coverage < 90%.

### Gene abundance analysis

Ensembl annotations were used to assign counts to reads aligned against the genomes. We considered reads that align with less than 5% of the sequence to an exon to be intergenic. Curated immune gene lists derived from Belov et al. 2007 [36] and Wong et al. 2006 [37] were specially used to derive read counts for immune genes. Fisher's exact test was used to test for differential expression between the two thymuses using the R statistical language [121]. Benjamini-Hochberg's method (FDR) [122] was used to correct for multiple testing.

Functional term enrichment analyses were performed using DAVID [123] and Ontologizer [124]. To obtain a more careful in depth look at genes of interest, we searched reads mapping to particular genes against the NCBI nr databases and also examined the genomic positions of reads using the UCSC genome browser by adding custom tracks to the opossum assembly.

### Targeted gene search

To check the presence of gene expression for genes that may have been excluded by the stringent filtering procedure, we used a less conservative method of removing reads of low quality. This involves the stripping of vectors and 454 adapters and excising repeat regions identified by RepeatMasker [125]. Reads were assembled using CAP3 [126]. Contigs aligning with E-value < 10^-5 ^were assigned to Ensembl gene models. Common genes between this gene list, the curated immune genes and genes identified for abundance analysis were determined by comparing gene symbols.

### Non-coding genes

Coding potentials of assembled transcripts were assessed using CPC [81]. Reads aligning < 100 kb from opossum Ensembl genes in the genome were removed. Potential non-coding contigs were searched using blastn against the UTRfull database, a comprehensive collection of human UTR sequences curated by UTRdb [83]. Those matching with E-values < 10^-3 ^were removed from the pool of potential nrRNA candidates. Remaining sequences were annotated by searching fRNAdb [84] using blastn (E-value cut-off < 10^-3^) and searching against the Rfam [85] covariance models.

### Tissue-specific antigens

Genes determined to be regulated by *Aire *in mouse medullary thymic epithelial cells from Affymetrix microarrays (Mouse 430 2.0) [115] were compared to wallaby genes. Genes that were significantly differentially expressed between the two thymuses were analysed.

## Authors' contributions

EW performed the analyses, participated in the design of the study and wrote the manuscript. AP designed scripts used for read analyses and participated in study design. AH designed and ran scripts for read analyses. ALH performed the assembly of reads. MR supplied the animals and advised on the study and revised the manuscript. CP advised on the study and revised the manuscript. JF collected the samples. RM advised on the study and revised the manuscript. RG provided sequencing technology and advised on the study. KB conceived of the study, participated in study design and revised drafts of the manuscripts. All authors approved the final manuscript.

## Supplementary Material

Additional file 1 **MA plot**. Average count of a gene between the two thymuses is shown on the x-axis and the count difference between the two thymuses for the same gene is shown on the y-axis. No obvious outliers are observed. Click here for file

Additional file 2 **Graph examining gene length bias using Ensembl opossum gene models**. Length of gene is shown on the x-axis and log_10_(P-value) on the y-axis. No length bias is evident. Click here for file
