# Supplementary material for: Transcriptomic analysis supports similar functional roles for the two thymuses of the tammar wallaby
Source: BMC Genomics. 2011 Aug 19;12:420. doi: 10.1186/1471-2164-12-420 (PMC3173455; doi:10.1186/1471-2164-12-420)
Supplement: Additional file 1 — MA plot. Average count of a gene between the two thymuses is shown on the x-axis and the count difference between the two thymuses for the same gene is shown on the y-axis. No obvious outliers are observed. [file 1471-2164-12-420-S1.DOC]

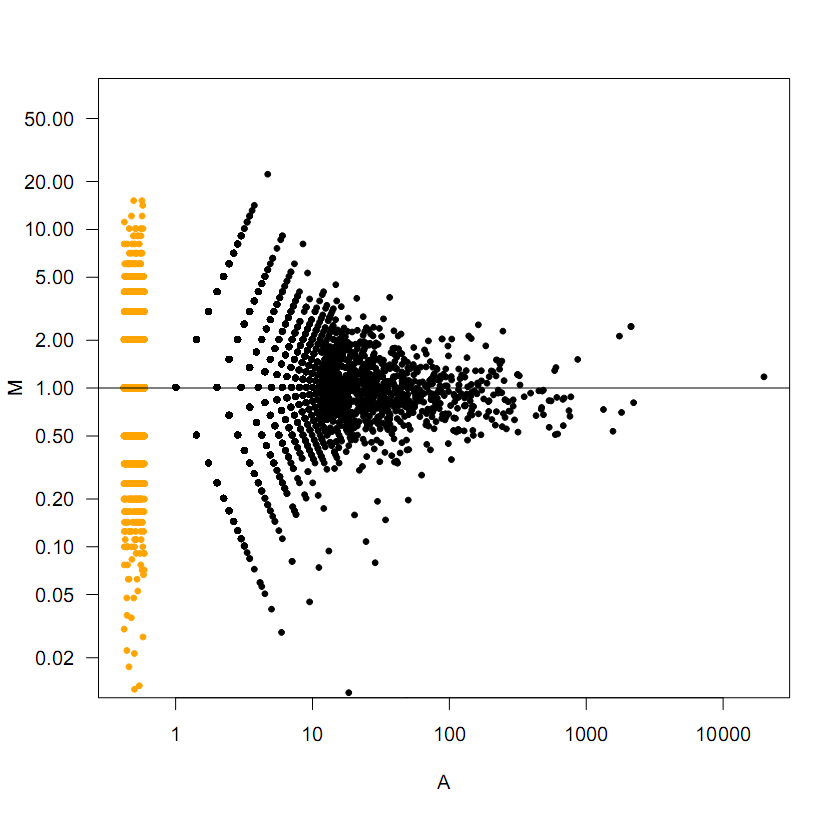


Additional file 1. A MA plot with the average count of a gene between the two thymuses on the x-axis and the count difference between the two thymuses for the same gene on the y-axis. No obvious outliers are observed.
