# Supplementary material for: Transcriptomic analysis supports similar functional roles for the two thymuses of the tammar wallaby
Source: BMC Genomics. 2011 Aug 19;12:420. doi: 10.1186/1471-2164-12-420 (PMC3173455; doi:10.1186/1471-2164-12-420)
Supplement: Additional file 2 — Graph examining gene length bias using Ensembl opossum gene models. Length of gene is shown on the x-axis and log10(P-value) on the y-axis. No length bias is evident. [file 1471-2164-12-420-S2.DOC]

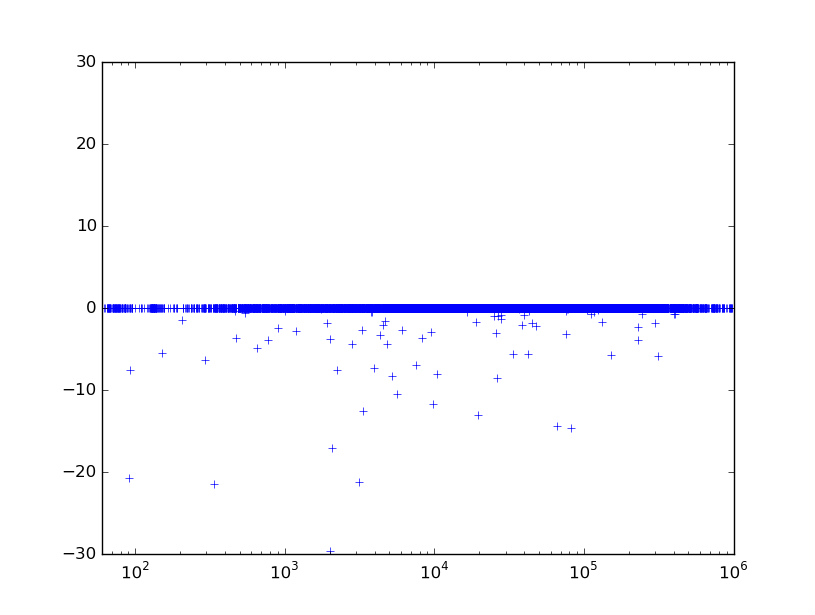


log_10_(P-value)

Gene length

Additional file 2. Graph examining gene length bias using Ensembl opossum gene models, showing length of gene on the x-axis versus log_10_(P-value) on the y-axis. There is no length bias.
